# Mapping loneliness prevention and alleviation interventions: a comparative survey of Basel, Bern, Zurich, and Dublin

**DOI:** 10.3389/fpubh.2026.1712906

**Published:** 2026-02-12

**Authors:** Stephen R. Milford, Roos Vernooij, Michael Rost, Charlotte Gehlen, Bernice S. Elger, B. Zara Malgir

**Affiliations:** 1 University of Basel Institut fur Bio- und Medizinethik, Basel, Switzerland; 2 North-West University, Potchefstroom, South Africa; 3 Institute of Midwifery and Reproductive Health, Zurich University of Applied Sciences, Winterthur, Switzerland; 4 Center for Legal Medicine, University of Geneva, Geneva, Switzerland

**Keywords:** loneliness, loneliness mapping, loneliness prevention, intervention mapping, social relationship expectation (SRE) framework, Switzerland, Ireland

## Abstract

**Introduction:**

Loneliness affects 38% of Swiss residents, higher than the global average. Considering that loneliness is associated with increased morbidity and mortality akin to smoking, drinking and obesity, the state of loneliness represents a serious health risk. To date no study has been undertaken to assess the landscape of loneliness prevention and alleviation interventions (LPAIs) in a high-income country like Switzerland and to compare this to other contexts.

**Results:**

NGOs delivered 84% of Swiss and 89% of Dublin LPAIs; direct state provision was ≤5%. Yet 60–75% of providers received some public funding, and 82% (CH) versus 93% (IE) were free to users. Older adults dominated addressees, while middle-aged adults, adolescents and chronically ill people were underserved. Support services and social-activity formats dominated, while evidence-based psychological interventions were scarce (<15%). LPAIs placed less focus on the Social Relationship Expectation variable Generativity, with Dublin LPAIs covering more Social Relationship Expectation domains and offering greater virtual access (78%) than Swiss counterparts (≤35%).

**Discussion:**

The landscape is rich yet fragmented. Heavy reliance on NGO delivery and ad-hoc funding jeopardises sustainability and equity. Under-representation of active, generative and tech-enabled formats signals possible low-cost missed opportunities for areas where loneliness is rising fastest. State leadership, strategic funding and digital innovation could close these gaps.

**Conclusion:**

High-income cities host many LPAIs, but without coordinated public-health strategies they fall short of preventive potential. Governments should mainstream, subsidise and modernise interventions to meet the multidimensional challenge of urban loneliness.

## Introduction

Loneliness and social isolation are serious health risks (1, 2). While social isolation refers to the objective social state of an individual (such as the frequency of social contacts) (3, 4), perceived social isolation, also known as loneliness (5), is defined as the subjective, unwelcomed feeling of a lack/loss of companionship (6). As a social determinant of health, loneliness has been consistently linked to poor physical and mental health outcomes. It is empirically linked to mental health deterioration (7); including moderate/high psychological distress and depression (8), as well as higher risk of paranoia and psychotic symptoms (2). Beyond mental health, loneliness has been causally connected with physical ailments. Research has demonstrated that people living with loneliness are more likely to report asthma, migraines, osteoarthritis, hypertension, and even back pain (9). It is associated with chronic diseases, high cholesterol levels, diabetes, and generally impaired self-perceived health. Consequently, people living with loneliness make more visits to medical doctors than people who do not feel lonely (8). Loneliness has been identified as a cardiovascular risk factor (1, 10), independently of smoking, hypertension or obesity, and is significantly associated with increased general morbidity and mortality, amounting to a relative increase of mortality risk of between 26 and 32% (11). This is comparable to the mortality risk of physical inactivity, smoking, alcohol consumption and obesity (10, 12–14). All this indicates that loneliness is an increased burden on state health resources. It is, therefore, no wonder that global organisations and countries alike have drawn attention to the link between health inequality, social isolation, and loneliness (8). This includes going so far as to assign ‘loneliness ministers’ in both the UK and (partially as a result of COVID-19) Japan (15, 16). Although it should be noted that the UK has demoted this ministry in recent years.

While loneliness is often referred to as a universal human experience (17), studies demonstrate distinct patterns across population groups and countries. Some studies point to a U-shaped distribution, with the highest levels of loneliness observed in people younger than 25 and older than 65 (18, 19). Other examples of at-risk groups include marginalised population groups [e.g., immigrants and sexual minorities (10)] and single persons (10).

About one-third of the world’s population experiences loneliness (20) and the number of lonely people is increasing (21–23). This trend is particularly evident in European countries, where rates of loneliness have doubled over recent decades (15). It has been argued that loneliness decreases with better education or higher income (24). However, a few countries are proving the opposite. Switzerland, for example, is one of the most prosperous countries in the world (25), and has a highly developed welfare system (26). Yet its population is reporting increasing loneliness. In 2005, 26% of the population reported feeling lonely “at least some of the time,” (27) by 2017 that percentage had risen to 38% (28)—surpassing the global average of 33% (20).

Overall, there is consensus that loneliness is not an immutable innate trait. Rather, it is malleable and can be exacerbated or ameliorated by active intervention (2, 9, 29–31). Numerous interventions have been recommended to alleviate loneliness: from social prescribing (16, 32), to technological interventions (33), from loneliness pills (34), and robots (10), to spontaneous singing (35). Other activities include social groups or mental health support programmes where these are specifically focused on loneliness. Together we term these activities: loneliness prevention and alleviation interventions (LPAIs).

Research on LPAIs is scarce. Often loneliness is grouped together with mental health and included in general mental health surveys. For example, Duncan et al. (36) identified 407 community-based, non-clinical mental interventions in England, of which 156 addressed loneliness. In very rare instances specific interventions are mapped across countries, such as befriending services in Scotland (37). A very limited number of survey’s have mapped interventions in in individual cities such as Barcelona, Spain (38), while a few directories—some analogue (39), some presented as interactive maps (40–42)—have been developed to assist those experiencing loneliness to identify interventions within a certain proximity. Importantly, a very recent Joint Research Centre (JRC) EU report, mapping loneliness interventions in the EU, has returned limited results (43). The report, focused solely on the 27 EU states, returned only 322 projects across 282 organisations. With a population of over 450 million people, this indicates only 2.7 interventions per million inhabitants.

Thus serious gapes exist as to the number, objectives, and types of LPAIs that are easily accessible to the public, the delivery and funding models for LPAIs, and the general cost of LPAIs to participants. This is especially true for countries like Switzerland and its major cities.

Some of these questions are addressed in this study, which conducted a survey of non-pharmaceutical LPAIs as part of a larger study on loneliness within the Swiss context aiming to investigate what interventions may be included in pandemic management plans to mitigate loneliness. During this project, three Swiss cities were surveyed in order to better understand the state of LPAIs in Switzerland. Here we present the results of this survey and our analysis into LPAIs within high-income, high-loneliness contexts. Uniquely, our analysis makes use of the social relationship expectation (SRE) framework as expounded on by Akther-Khan et al. (44) This framework recognises the subjective experience of loneliness and the discrepancy between the expected and actual quality of social relationships based on six expectation aspects that need to be fulfilled in order to meet a person’s social relationship expectations. These expectation aspects are as follows: 1) Proximity (having social relationships available), 2) Support (feeling cared for and able to rely on others), 3) Intimacy (feeling close, listened to, and understood by others), 4) Fun (sharing interests and enjoyable experiences with others), 5) Generativity (having opportunities to contribute meaningfully to others), 6) Respect (feeling valued and activity included in society). Using this framework, researchers were able to more objectively evaluate LPAIs possible suitability for decreasing the subjective feeling of loneliness among participants.

## Methodology

The aim of the survey was to identify, catalogue, and evaluate the state of LPAIs available and accessible to the public in the three largest German-speaking Swiss cities (Basel, Bern, and Zurich) and to compare these findings with a similar, non-Swiss context. Dublin was chosen as the comparison city as Ireland’s per capita GDP is comparable to Switzerland’s ($104 K vs. $100 K, respectively), although there is a difference in population size, Dublin’s population being approximately twice the size of all three Swiss cities combined [1.4 M compared to Basel’s 173 K (45); Bern’s 134 K (46); and Zurich’s 423 K (47)], Importantly, recent work by the European Commission’s Joint Research Centre demonstrated that Ireland has the highest prevalence of loneliness of all European countries (48).

### Data collection

This research adopted the vantage point of a member of the public experiencing loneliness and actively seeking accessible support. A key guiding question was: What would a person reasonably do if they were trying to access an LPAI in their city? Two primary actions were identified: (1) searching online (via search engines or social media), and (2) speaking to a professional or key community member likely to be aware of local LPAIs (e.g., social worker, psychologist, government official etc.).

To address activity 1, a semi-structured search of online sources was undertaken. This involved developing a search string with input from an experienced information specialist and searching the two largest internet search engines (Google and Bing), as well as two popular social media platforms suitable for groups (Facebook and Instagram). For the search engines, the search string included the relevant city (e.g., Basel) and 30 terms related to concepts such as loneliness, social isolation, social activity, intervention, club etc. presented in German for the Swiss searches and English for the Irish search (see Appendix for the full search string). Using Google Chrome’s Incognito feature, the search string was inputted into Google and Bing using the advanced search feature. The first two search terms (e.g., ‘Basel’ and ‘Einsam*’ [Lonely]) were entered into the ‘Find pages with all these words’ search field, while the rest of the search string was included in the field labelled ‘Find pages with any of these words.’ Results from each search engine were subjected to abstract and title screening. As it was not possible to conduct the screening in one sitting, and because search engines return different results upon each search query, the search was run five times per engine. For each run, the first 200 results (20 pages) of titles and abstracts were screened, yielding a total of 2000 screened entries per city (8,000 in total). This number was deemed appropriate to avoid missing less visible or poorly indexed LPAIs, and was considered beyond what a reasonable member of the public would consider when seeking for an intervention online. Any website title or abstract that was deemed to potentially adhere to the inclusion criteria was bookmarked for full screening (nr. 397). In addition, an unstructured search was conducted on two popular social media platforms—Facebook and Instagram—suitable for LPAIs (i.e., platforms that enable or promote the formation of groups). A researcher searched these platforms for groups, organisations, or interventions that might offer LPAIs in the survey cities, and attempted to contact them through the platform or via their website where this was identifiable. This search resulted in seven LPAIs identified.

**Figure 1:**
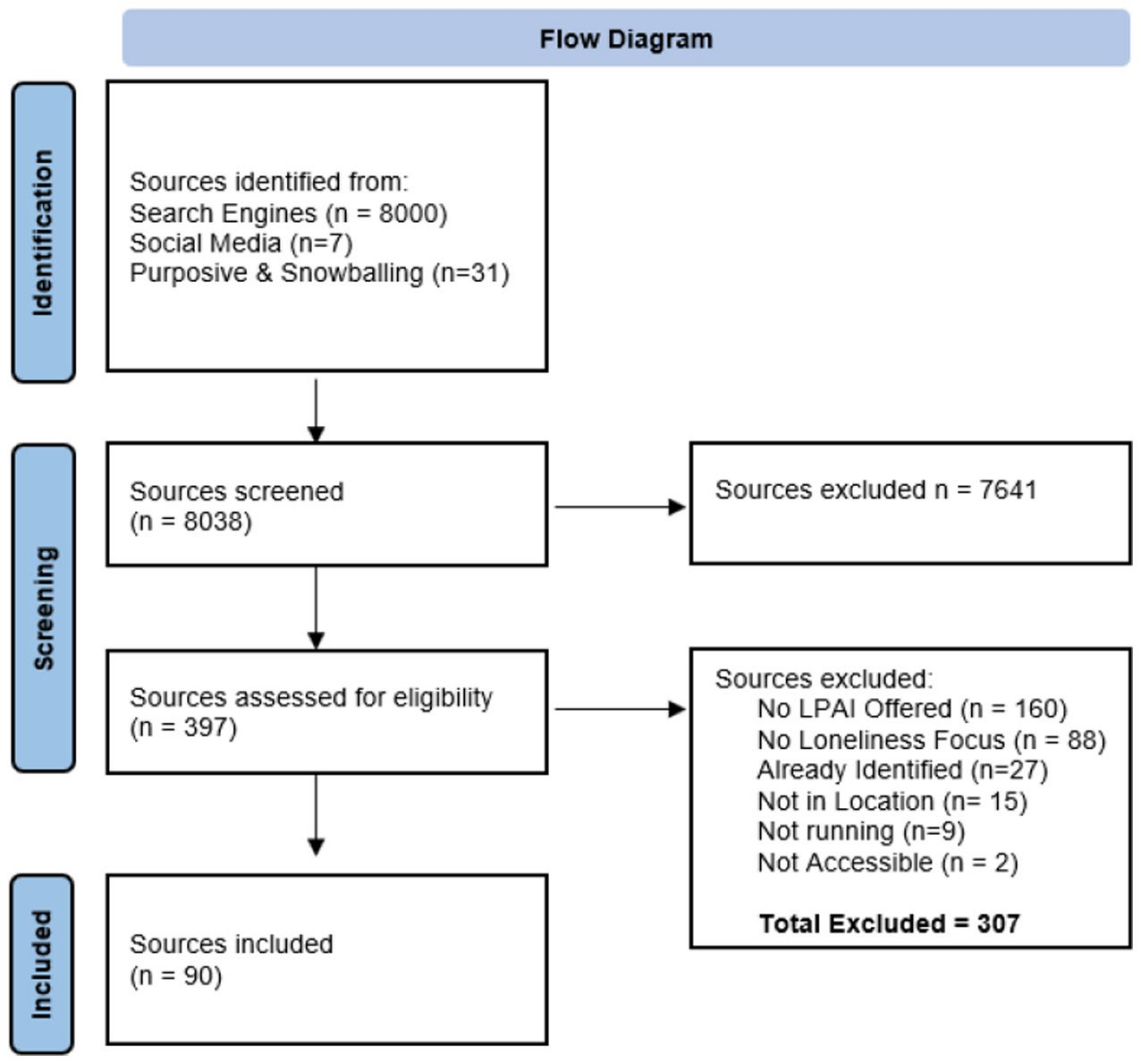
PRISMA inspired flow diagram detailing entries identified through semi-structured searches of internet search engines and social media platforms as well as through purposive and snowball recruiting.

Alongside the semi-structured search, and to address activity 2, researchers engaged in purposive and snowball sampling. A list of key community members who may have knowledge of LPAIs available in the respective cities was developed. This included social workers, officials at health department, or locally active politicians. To avoid contacting every physician, social worker or politician, only senior members or those directly responsible for relevant services such as mental health (e.g., the head of the department of health or social affairs in a Swiss canton) were contacted by email at first. Key members were asked for details of possible LPAIs as well as the names of other key community members who may be further aware of LPAIs in each city. seventy-six key members were contact (of which 31 LPAIs were identified through this sampling methodology, many being duplicates of LPAIs identify in the semi-structured search of internet search engines and social media platforms. In total, 90 sources were included in this study (Figure 1).

Sources were included if they met the following criteria: they included an LPAI (i.e., intervention/support accessible to someone experiencing loneliness); were located in the respective city (e.g., Basel) or were easily accessible from the respective city if virtual (e.g., a national telephonic helpline like 143.ch); explicitly focused on the prevention or alleviation of loneliness (e.g., mentioned loneliness on their website, in reports, or through blogs/articles about loneliness alongside their offerings); and if the LPAI was currently active or had ceased operations within the past 2 years.

Sources were excluded if they met one or more of the following exclusion criteria: they were not located in the respective city; the website/organisation/LPAI had no clear or discernible focus on loneliness;^1^ the website was purely informational without offering an activity/intervention accessible to someone experiencing loneliness (e.g., online newspaper articles about loneliness in a city), the content was solely oriented towards entertainment or leisure; the LPAI was aimed at professionals rather than end-users (e.g., training courses on loneliness alleviation for social workers); or ceased more than 2 years before data collection started (i.e., before June 2024). Data collection ended in June 2025. Sources that did not meet the inclusion criteria were excluded from further consideration, while included sources were subjected to data extraction.

All identified organisations/LPAIs were contacted and requested to complete an online survey. Surveys aimed to offer LPAIs the opportunity to clarify or correct publicly available information obtained through our semi-structured search as well as to enable LPAIs to provide us with information that was not on their website but remained publicly available (e.g., through an LPAI’s printed pamphlet) The survey, designed on Qualtrics.com (an online platform suitable for qualitative and quantitative data collection), was closely aligned with the data extraction sheet and included clear instructions to avoid any private or sensitive data and to only include publicly available data. The survey was available in both English and German, and included 24 questions taking approximately 15 min to complete. The survey was internally pilot tested with members of the research institute before being launched. Twenty-five surveys were completed in German and six completed in English. Where discrepancies between information obtain via an LAPI’s website and a completed survey were identified, completed surveys took president. Respondents were also asked whether they knew of any other LPAIs that might meet the study’s inclusion/exclusion criteria.

Ethical review and approval for this study were obtained from the Ethics Committee of the Department of Psychology at the University of Basel (Application number: 036-23-2) as part of the INCLUDE project. Data Extraction and Coding.

The following attributes from the included sources were extracted and stored in an Excel sheet: LPAI/organisation name, website, contact details, organisational structure, parent organisation, multi-city availability, language, funding source, cost to participants etc. The first and last authors coded the data by ascribing a code to each attribute applicable to the included sources (e.g., Intended Beneficiary may be ascribed AD for adolescents, YA for young adults, OA for older adults etc.). Coded sections included data such as the type of LPAI offered, its objective, the duration, or the SRE score. For highly subjective attributes—such as the type and objective of the LPAI—a Cohen’s kappa was calculated to assess interrater reliability. A Kappa of 0.805 was achieved, indicating good inter-coder agreement (49).

### Data analysis

The extracted and coded data was subjected to descriptive statistical analysis. First, the total number of interventions and the number of codes present for each item category was calculated. For all results presented below, except those pertaining to the SRE score, absolute numbers were converted to percentages. Absolute numbers were used to assess the SRE score.

A deductive analysis was conducted to assess whether, and how, LPAIs in the surveyed cities addressed the six aspects of the SRE framework as expounded on Akther-Khan et al. (44) Researchers met to align on the interpretation of each SRE aspect and to code a large section of the data together. Each for each aspect of the SRE a binary ‘yes’ or ‘no’ score was given (‘yes’ = present; ‘no’ = not present). Researchers documented written justifications for each decision. Regular meeting we held to ensure that coding of the SRE remained consistent throughout data analysis. The deductive analysis offered researchers insights into how many LPAIs in each surveyed city either included or excluded aspects of the SRE framework and thereby their overall ability to address participants subjective social relational expectations.

## Results

### Structure, funding sources, and costs

**Figure 2:**
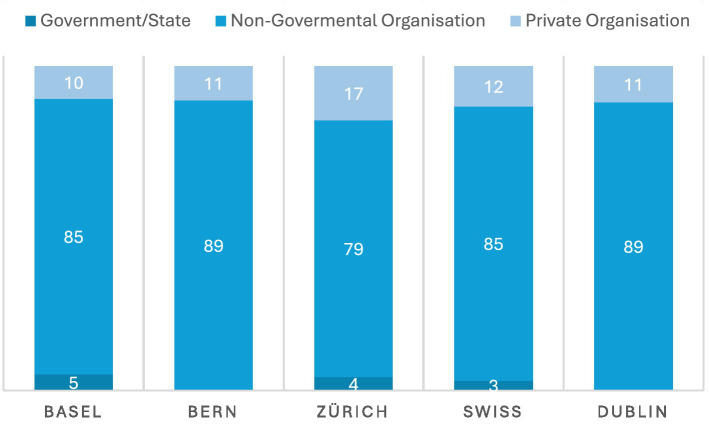
Organisational structure. Across all surveyed cities, NGOs are by far the dominant providers of LPAIs (around 80–90%), with private organisations forming a small minority and direct state-run provision being rare or absent.

Non-Governmental Organisations (NGOs) accounted for between 80 and 90% of the organisations responsible for activities addressing loneliness in the surveyed cities (see Figure 2). For Bern and Dublin the number was as high as 89%, while Zurich was slightly lower at 79%, with a Swiss average of 84%. Private organisations (such as care facilities or therapy practices) accounted for between 10 and 17% of interventions, with the Swiss average being 13%, comparable to Dublin’s 11%. A very small percentage of LPAIs were solely organised by the state (5% in Basel and 4% in Zurich). In Dublin and Bern we were unable to find an LPAI solely organised by the national or cantonal government (Table 1).

**Table 1:** Structure, funding sources, and costs.

LPAI/organisation name	Organisational structure	Funding sources	Cost to participants (in CHF)
Basel			
Begegnungszentrum CURA	NGO	PF	Free
Bestcompanion	PR	PF	$$$
BewegungPlus Basel	NGO	PF	Free
Caritas	NGO	PF/CR/SF/MF	Free
Dovida	PR	PF	$$$$
Fundus Basel	NGO	PF/SF/CR/MF	Free
Grauepanther	NGO	PF	$$
Häschziit	NGO	SF/PF/CR/MF	Free
JuBe Basel	NGO	SF/PF/MF	Free
Jugendzentrum Dreirosen	NGO	SF/CR/MF	Free
M-Eating Table	NGO	COR	Free
Mein Ohr für Dich	NGO	PF/CR/MF	Free
NotAlone im Quartier	NGO	CR/SF/MF	Free
Plauderbank	GOV	SF	Free
Plauderkasse	NGO	SF/PF/CR/MF	Free
Pro Senectute	NGO/PR	SF/PF/CR/MF	Free/$/$$/$$$/$$$$
Schweizerisches Rotes Kreuz Basel	NGO	CR/PF/MF	Free
Treffpunkt Glaibasel	NGO	PF/CR/SF/MF	Free
ZämmehAlt	NGO	PF/CR/SF/MF	Free
Zentrum Selbsthilfe	NGO	PF/CR/SF/MF	Free/$$$
Bern			
Caritas	NGO	SF/PF/CR/MF	Free/$$
Connect! - Together Less Lonely	NGO	CR	Free
CONTIGO	NGO	SF/CR	Free
Coontact	NGO	MF	Free
Der Besuchdienst	NGO	PF/SF/CR/MF	$$/$$$
Graue Panther Bern	NGO	PF	Free/$/$$$$
Heilsarmee	NGO	PF/CR/SF/MF	Free/$/$$
Helsana	PR	PF/CR/MF	$$$$
Malreden	NGO	PF/CR/SF/MF	Free
Nachbarschaft Bern VBG	NGO	SF	Free
Netzwerk Erzählcafé	NGO	MF	Free
Offene Kirche Bern	NGO	PF/SF/CR/MF	Free/$$
Pro Infirmis	NGO	SF/PF/CR/MF	Free/$$
Pro Senectute Bern	NGO	SF/PF/CR/MF	Free/$$$
Schweizerisches rotes Kreuz Bern	NGO	SF/PF/CR/MF	Free/$$$
Selbsthilfe Bern	NGO	SF/PF/CR/MF	Free/$$
Soli Bern	NGO	PF	Free
Spitex Bern	NGO	PF/SF/CR/MF	$/$$
Tavolata	PR	PF/CR/MF	Free/$/$$
Zürich			
147.ch	NGO	SF/CR/PF/MF	Free
143.ch	NGO	SF/CR/PF/MF	Free
Anton Schumann Coaching Zurich	PR	PF	$$$$
Arche Zurich	NGO	PF	Free
Café Yucca	NGO	SF/CR/PF/MF	Free
Caring Communities	NGO	SF/CR/PF/MF	Free
Caritas	NGO	SF/CR/PF/MF	Free
Räber - Coaching & Persönlichkeitsentwicklung	PR	PF	$$$$
Einsamkeit im Alter	NGO	SF/CR/PF/MF	Free
Gesundheitszentren für das Alter	GOV	SF/PS	$$$/$$$$
Insieme Zürich	NGO	SF/PF	Free
Katholisch Kirche Stadt Zürich	NGO	SF/CR/PF/MF	Free
Netz4	NGO	SF/CR/PF/MF	Free
Pro Mente Sana	NGO	SF/CR/PF/MF	Free
Pro Senectute	NGO	SF/CR/PF/MF	Free
Psyvita	PR	PF	$$$$
Reformierte Kirche in Zürich	NGO	SF/PF	Free
Schweizerisches Rotes Kreuz Kanton Zürich	NGO	SF/PF/CR/MF	Free
Seelsorge.net	NGO	CR/PF/MF	Free
Selbsthilfe Schweiz	NGO	SF/CR/PF/MF	Free
Solino	NGO	SF/CR/MF	Free/$
Sozialkontakt	NGO	PF	Free/$$$
Spitex Pflege Zürich GmbH	PR	SF/CR/PF/MF	$$$$
Wie geht’s dir?	NGO	SF	Free
Dublin			
All Ireland Social Prescribing Network	NGO	SF/CR/MF	Free
Alone.ie	NGO	SF/CR/PF/MF	Free
Archdiocese of Dublin	NGO	SF/CR/PF/MF	Free
Aware.ie	NGO	SF/CR/PF/MF	Free
Belongto	NGO	SF/CR/PR/MF	Free
Childline	NGO	SF/PF/MF	Free
City Therapy	PR	PF	$$$$
Community Development Team	NGO	SF/MF	Free
Cross Care	NGO	SF/PF/CR/MF	Free
Dublin City Community Cooperative	NGO	SF	Free
Friends of the Elderly	NGO	CR/PF/MF	Free
Grow Mental Health	NGO	SF/PF/MF	Free
Haven Hub	NGO	SF/PF/CR/MF	Free
Jigsaw	NGO	SF/PF/CR/MF	Free
Making Connections	NGO	PF/CR	Free
Men’s Sheds	NGO	SF/PF	Free
Mind and Body Works	PR	PF	$$$/$$$$
Parentline	NGO	SF/PF/MF	Free
Samaritans	NGO	SF/CR/PF/MF	Free
Seniorline	NGO	SF/PF/MF	Free
Shine	NGO	SF/PF/MF	Free
Social Prescribing	NGO	SF/CR/MF	Free
SOSAD Ireland	NGO	PF/CR/MF	Free
SpuNout	NGO	SF/PF/MF	Free
Barnardos	NGO	SF	Free
The Crafty Ladies	NGO	CR/PF/MF	Free
Thrive	PR	CR	Free

GOV, Government/State Run; NGO, Non-Governmental Organisation; PR, Private Organisation; “$, < 10; $$, 10–30; $$$, 30–50; $$$$, > 50; SF, State Funding; CR, Corporate/Foundation Funding; PF, Public Donation Funding; MF, Mixed Funding.

**Figure 3:**
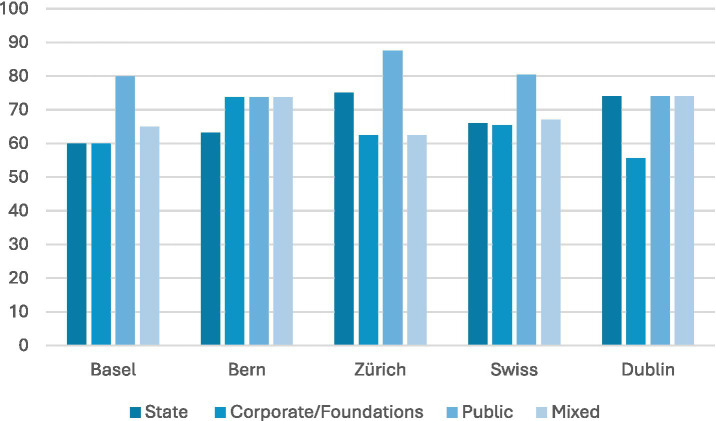
Funding sources. Most LPAIs rely on public donations and some state funding (state funding present in ~60–75% of organisations; public donations in ~74–88%), Dublin shows relatively higher state funding but lower corporate/foundation support.

This is not to say that the state remains uninvolved. Although it was not possible to ascertain the proportion of funding individual LAPIs received from the state, the data was able to identify the vast majority of organisations received some state funding as part of their funding structure (see Figure 3). This ranged from 60% of organisations in Basel to up to 75% or 74% for Zurich and Dublin respectively, with a Swiss average of 66%. LPAIs in Dublin, however, were on average more likely to receive state funding than Swiss LPAIs (74% vs. 66%), yet less likely to receive corporate sponsorship or funding from non-profit foundations (56% compared to the Swiss average of 65%). Dublin also had a lower percentage of organisations being supported by public donations (74%) than the average Swiss city (80%), possibly due to its higher state funding. Nevertheless, it should be noted that the vast majority of LPAIs were funded at least partly by public donations (ranging from as low as 74% in Bern, to as high as 88% of LPAIs in Zurich).

**Figure 4:**
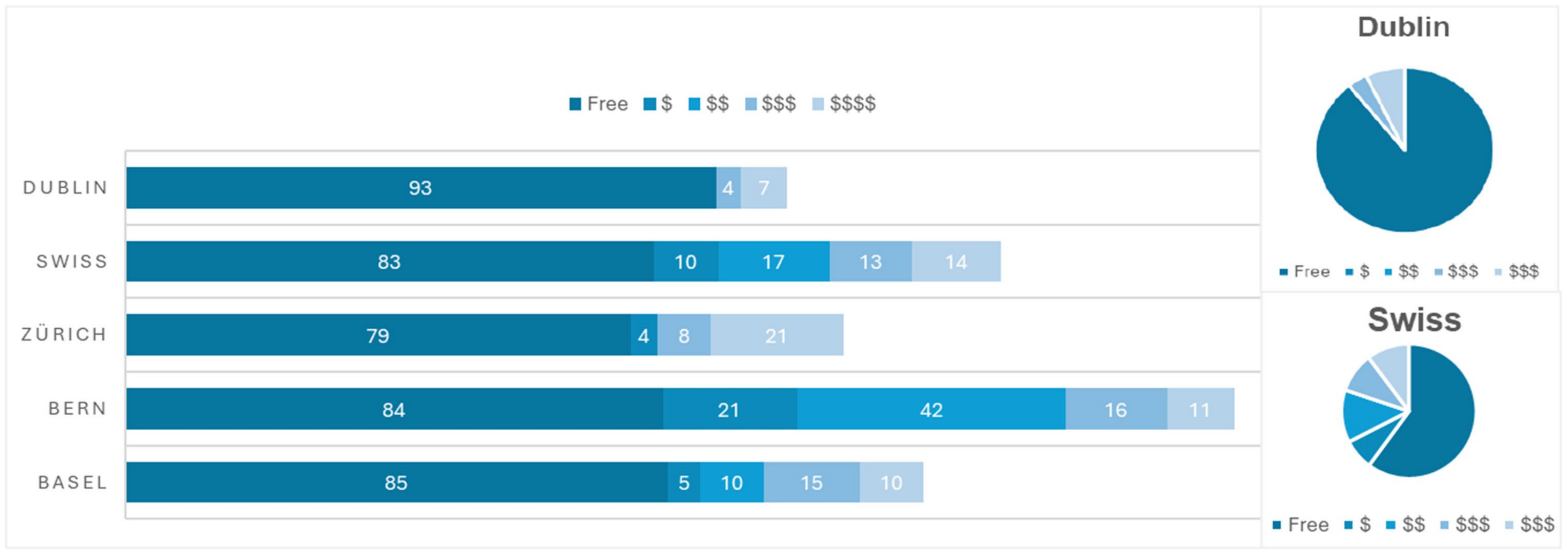
Costs to participants. Most LPAIs are free (Swiss avg. 82%; 79–93% by city), while paid services are often very expensive (>50 CHF), peaking in Zurich (22%) while Dublin had more paid LPAIs than Swiss cities on average.

A wide disparity was observed in the costs borne by participants engaging with an LPAI (see Figure 4). A very high proportion of LPAIs were offered free of charge to participants (ranging from 79% in Zurich to 93% in Dublin, with a Swiss average of 82%). A small number of LPAIs were considered very expensive (>50 CHF) for participants to engage with (ranging from 7% of LPAIs in Dublin to as high as 22% of LPAIs in Zurich). These were mostly private organisations such as care centres, or therapy and coaching services. Many organisations offered various different LPAIs with differing costs to participants, ranging from free to very expensive services. For example, Pro Senectute of the two Basel cantons (“Beider Basel”) mentioned on their website a free support hotline, but individuals were charged for participating in courses, while Sozialkontakt (Zurich) allowed individuals to join their website as free “basic members,” or they could upgrade to a premium membership—available for a yearly or half-yearly fee—which granted access to additional features on the website such as the ability to send unlimited contact requests. Bern represented the biggest spread (84% free, 21% low cost, 42% medium, 16% higher cost and 11% very expensive). LPAIs in Dublin were more likely to be either free or expensive (>50 CHFs) than be cheaper or moderately expensive (between 10 and 50 CHFs). One could conclude, on this data, that in Dublin one is more likely to access free LPAIs, but if one is going to pay for an LPAI, it is likely to be more expensive than in Switzerland. Notably, the higher-cost LPAIs were disproportionately delivered by private providers (e.g., therapy/coaching practices and some care services). This suggests that the most structured forms of support may be less financially accessible than the predominantly free NGO-led offerings.

### Intended beneficiaries, group size, and setting

**Figure 5:**
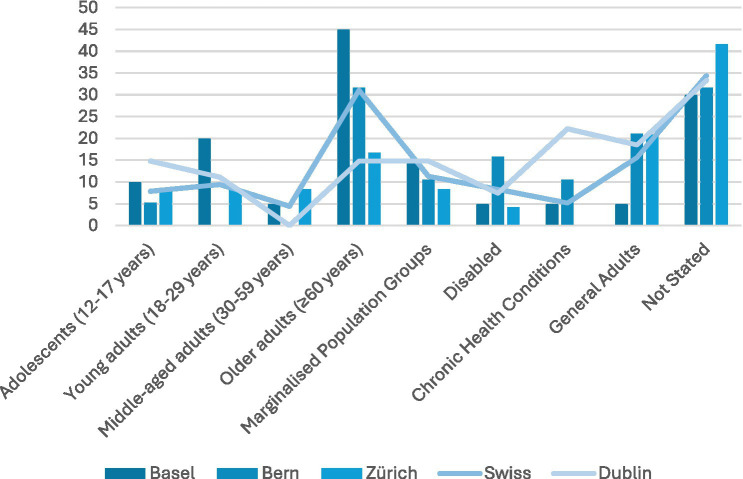
Intended beneficiaries. Older adults were the main target of LPAIS (Basel 45%, Bern 32%, Zurich 17%), while middle-aged adults were rarely targeted (Swiss avg. 4%; none in Dublin); marginalised groups received moderate focus (8–15%).

While the majority of LPAIs had not clearly stated their intended audience, and thereby were assumed to have a wide catchment field, a large proportion of LPAIs surveyed focused on older adults (above 60 years’ old; see Figure 5). This was particularly true in Basel, where 45% of LPAIs were designed for older adults, with Bern and Zurich having 32 and 17%, respectively. A second well-served group was that of marginalised populations (e.g., immigrants, or sexual minorities) with 15, 11 and 8% of LPAIs in Basel, Bern and Zurich, respectively, focused on marginalised populations compared to Dublin’s 15%. Few LPAIs focused on adolescence, disabled persons, or those living with chronic illness—all well-established risk groups. Middle-aged adults (30–59 years old) were particularly underserved with a Swiss average of only 4% of LPAIs and no LPAIs identified in Dublin focused only on this group. However, it should be noted that a number of interventions were addressed at the general adult population (5% in Basel, 21% in Bern, and 21% in Zurich, compared to 19% in Dublin) and middle-aged adults would be included in this group.

**Figure 6:**
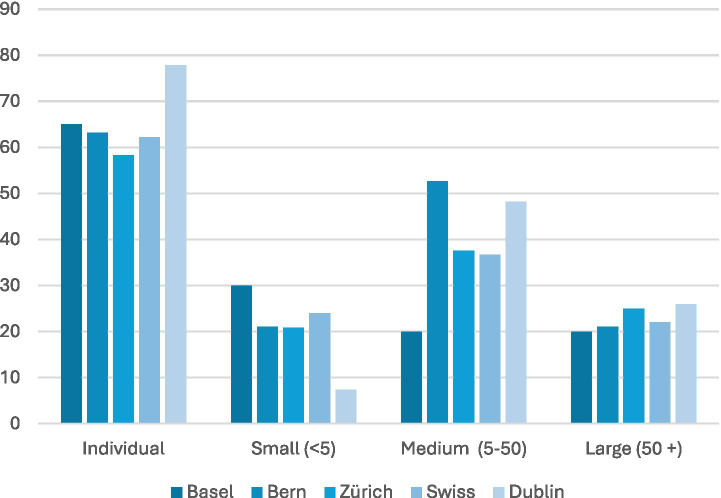
Delivery format by group size. Most LPAIs were aimed at individuals (up to 78% in Dublin), with many designed for medium groups (10–50) in Bern/Dublin (~53%/48%); small (<10) and large (>50) formats were uncommon.

The majority of LPAIs in our survey were focused on individuals, reaching as high as 78% of LPAIs in Dublin (see Figure 6). This includes, for example, one-on-one therapy sessions, or programmers in Basel like Plauderkasse (where individuals could spend more time at the checkout chatting to a cashier), or Plauderbank (where individuals might sit on a public bench dedicated to chatting with a volunteer about loneliness). Many LPAIs were focused on medium-sized groups (10 to 50 people), with 50% of those surveyed in Bern and Dublin tailored to groups of this size (53 and 48%, respectively). Such interventions could be, for example, Zentrum Selbsthilfe in Basel (The Centre for Self-help) that brought youth together in moderated medium sized groups, or the organisation for older adults called “Graue Panther” (Grey Panther) in Bern which often ran events (talks, presentations, social activities etc.) for groups between 10 and 50 participants. Far fewer LPAIs were focused on smaller group sizes (>0 participants) or larger groups (<50 participants), with Dublin offering only 7% LPAIs tailored to groups smaller than 10 (Table 2).

**Table 2:** Intended beneficiary, group size, and setting.

LPAI/organisation name	Intended beneficiary	Participant size	Technology usage	Location
Basel				
Begegnungszentrum CURA	CHC/YA/MA/OA	M	NT	PR
Bestcompanion	OA	I	NT	PR
BewegungPlus Basel	NS	S-M	PC/VC/SM/STM	PS/PR/VS
Caritas	MP	I-L	PC/EM	PS/PR/VS
Dovida	OA	I	NT	PR
Fundus Basel	OA	I-M	NT	PS/PR
Grauepanther	OA	S-L	NT	PS
Häschziit	YA	I	SM/WP	VS
JuBe Basel	AD/YA	I	PC/VC/STM	PS/VS
Jugendzentrum Dreirosen	AD/YA	L	NT	PS/PR
M-Eating Table	NS	I	NT	PS
Mein Ohr für Dich	NS	I	PC	VS
NotAlone im Quartier	NS	I-S	APP	VS
Plauderbank	NS	S	NT	PS
Plauderkasse	NS	I	NT	PS
Pro Senectute	OA	S-L	NT	PS/PR
Schweizerisches Rotes Kreuz Basel	OA/MP/DA	I-S	NT	PS/PR
Treffpunkt Glaibasel	MP/OA	I	NT	PS
ZämmehAlt	OA	I	NT	PS/PR
Zentrum Selbsthilfe	GA	M	VC	PR/VS
Bern				
Caritas	MP	I-M	PC	PS/PR/VS
Connect! - Together Less Lonely	OA	S-L	PC/VC	PS/VS/PR
CONTIGO	NS	I-S	NT	PS/PS
Coontact	NS	I	PC/EM	VS
Der Besuchdienst	NS	I	NT	PS/PR
Graue Panther Bern	OA	M	NT	PS/PR
Heilsarmee	NS	I-L	PC	PS/PR/VS
Helsana	GA	I	PC	VS
Malreden	OA	I	PC	VS
Nachbarschaft Bern VBG	GA	M-L	PC	PS/PR/VS
Netzwerk Erzählcafé	GA	M	NT	PS
Offene Kirche Bern	AD/GA/MP	I-M	NT	PR
Pro Infirmis	DA	I	PC/STM/EM	PS/VS
Pro Senectute Bern	OA	I-M	NT	PS/PR
Schweizerisches rotes Kreuz Bern	OA	I-M	PC	PR/VS/PS
Selbsthilfe Bern	DA/CHC	S-M	VC	PS/VS/PR
Soli Bern	NS	M-L	NT	PS
Spitex Bern	OA/DA/CHC	I	NT	PR
Tavolata	NS	S-M	NT	PR
Zürich				
147.ch	AD	I	PC/STM/EM	VS
143.ch	GA	I	PC/STM/EM	VS
Anton Schumann Coaching Zurich	GA	I	VC	PR/VS
Arche Zurich	YA/MA	M	NT	PS
Café Yucca	NS	M	NT	PR
Caring Communities	NS	L	VC/SM	VS/PS
Caritas	MP	I-L	PC	PS/PR/VS
Räber - Coaching & Persönlichkeitsentwicklung	GA	I	EM	PR/VS
Einsamkeit im Alter	OA	S-L	NT	PS/PR
Gesundheitszentren für das Alter	OA	I	NT	PS/PR
Insieme Zürich	DA	M	NT	PR/PS
Katholisch Kirche Stadt Zürich	NS	I-M	NT	PR
Netz4	AD/YA/MA/MP	S-L	NT	PR
Pro Mente Sana	NS	I-M	PC/EM/VC	PR/PS/VS
Pro Senectute	OA	S-L	NT	PS/PR
Psyvita	GA	I	PC/VC	PR/VS
Reformierte Kirche in Zürich	NS	S-L	NT	PR
Schweizerisches Rotes Kreuz Kanton Zürich	NS	I-M	NT	PS/PR
Seelsorge.net	GA	I	PC/VC	VS
Selbsthilfe Schweiz	NS	I-M	VC	PS/PR/VS
Solino	NS	M	NT	PR
Sozialkontakt	NS	S-M	STM/WP/PC	PS/PR/VS
Spitex Pflege Zürich GmbH	OA	I	NT	PR
Wie geht’s dir?	NS	I	WP/SM/APP	VS
Dublin				
All Ireland Social Prescribing Network	GA/DA/MP/CHC	I-L	PC	VS/PS
Alone.ie	OA	I	PC	VS/PR
Archdiocese of Dublin	NS	M-L	NT	PR
Aware.ie	CHC	I-M	PC/VC/EM	PR/VS/PS
Belongto	AD/MP	I-L	PC	PR/VS
Childline	AD/YA	I	PC/STM/WP	VS
City Therapy	GA	I	VC	PR/VS
Community Development Team	NS	M-L	NT	PS
Cross Care	NS	I-M	PC/VC/STM	PS/PR/VS
Dublin City Community Cooperative	MP/DA/CHC	I-L	NT	PS/PR
Friends of the Elderly	OA	I-M	PC	PS/VS/PR
Grow Mental Health	GA	S-M	VC	PS/VS/PR
Haven Hub	NS	I	PC	PR/VS
Jigsaw	AD/YA	I-M	STM/WP	PR/VS
Making Connections	OA	I-M	PC/VC	PS/VS/PR
Men’s Sheds	M	M-L	NT	PR
Mind and Body Works	NS	I	VC	PR/VS
Parentline	NS	I	PC	VS
Samaritans	NS/CHC	I	PC/EM	VS
Seniorline	OA	I	PC	VS
Shine	CHC	I-M	PC/STM	PR/VR
Social Prescribing	GA	M-L	PC/VC	VS/PS
SOSAD Ireland	CHC	I-M	PC/VC	VS
SpuNout	AD/YA	I	STM/WP/EM/PC	VS
Barnardos	NS	I	EM/PC	PR/VS
The Crafty Ladies	MP/NS	S-M	NT	PR
Thrive	GA	I	VC	VS/PR

AD, Adolescents (12–17 years); YA, Young Adults (18–29 years); MA, Middle-aged Adults (30–59 years); OA, Older Adults (≥60 years); MP, Marginalized Populations—e.g. racial/ethnic minorities/LGBTQAI+; DA, People with Disabilities; CHC, People with Chronic Health Conditions; GA, General Adults; NS, Not Specified; I, Individual; S, Small (<10 participants); M, Medium (10–50 participants); L, Large (>50 participants); PC, Phone Calls; VC, Video Calls; SM, Social Media; STM, Short Messaging platform; WP, Webpage; APP, Phone/Web App; EM, Email; NT, No Technology Involved; PS, Public Setting; PR, Private Setting; VS, Virtual Setting; NK, Not Known.

**Figure 7:**
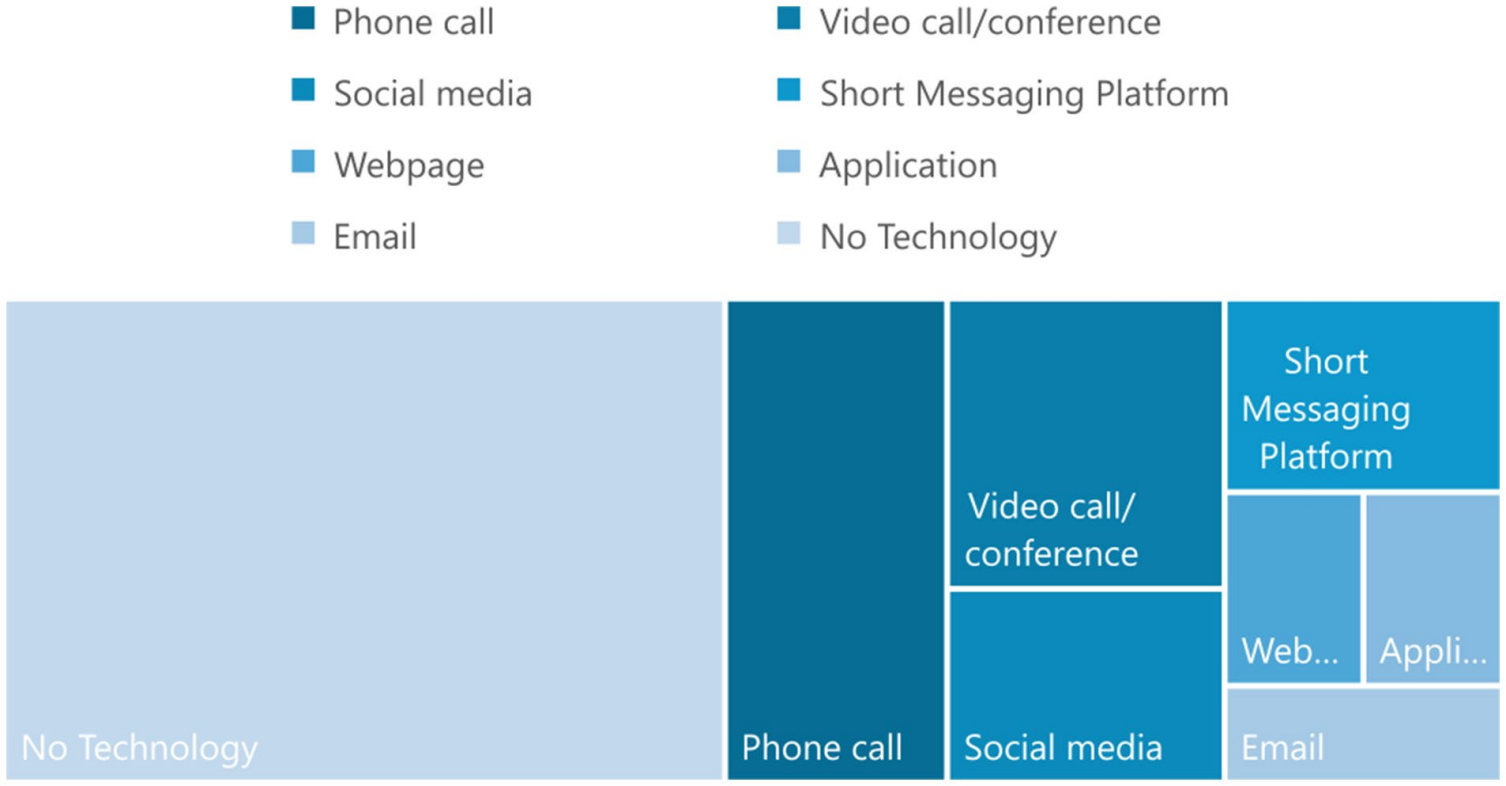
Use of technology. Swiss LPAls mostly referenced no technology (Basel 65%, Bern 47%, Zurich 50%), whereas Dublin was more digitally/virtually accessible (only 19% referencing no technology). Phone-based LPAls were dominant.

For the majority of Swiss LPAIs, technology (e.g., telephone, short messaging services such as WhatsApp, email etc.) played no role in their offerings (65% of LPAIs in Basel, 47% in Bern, 50% in Zurich; see Figure 7). These LPAIs focused on face-to-face contact and included many senior citizen focused organisations, visiting and accompanying services—such as Der Besuchsdienst in Bern –, or respite centres. The picture was entirely different for Dublin, where only 19% of LPAIs made no reference to technology.

The use of telecommunication services, such as phone calls, remained the most widely used form of technology for all LPAIs, ranging from 29% of interventions surveyed in Zurich, to as high as 63% of those surveyed in Dublin (see Figure 7). In many cases, LPAIs were solely accessible through the telephone, e.g., 147.ch, 143.ch, Malreden (Let us Talk), or Mein Ohr für Dich (My Ear for You). Overall, a good number of LPAIs were accessible in a virtual setting (e.g., by phone, or over video conference services such as through Zoom, as opposed to in person meetings). Basel, however, had the smallest percentage of LPAIs taking place virtually (35%, compared to Dublin’s 78%). A very small number of LPAIs (5% in Basel, 4% in Zurich) made extensive use of technology such as web/mobile phone-based applications. This included Wie geht’s dir? (How are you?) which employed a web-based and mobile phone application to assist participants to improve mental health, or the NotAlone im Quartier (NotAlone in the Neighbourhood) which employed an App to connect people living alone with a team to assess and assist their level of loneliness.

**Figure 8:**
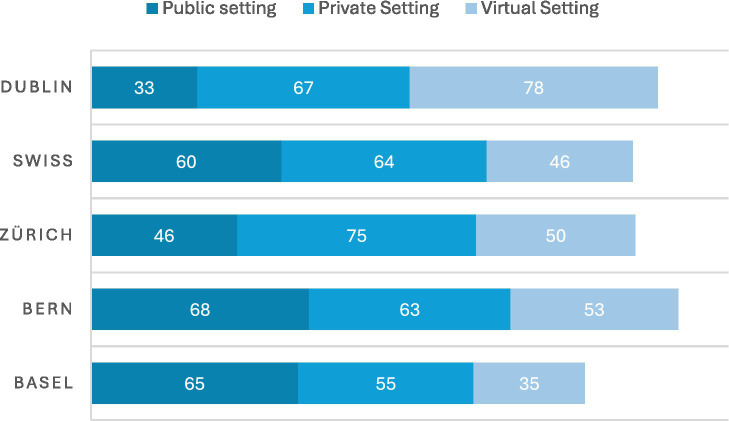
Location (setting). Zurich and Dublin favoured private premises (75 and 67%); while Basel and Bern based LPAIS mostly used state-owned venues (65 and 68%); Dublin had the lowest state-venue share (33%).

In-person LPAIs were most likely to take place on private property (e.g., a church building, or NGO’s premises) in Zurich and Dublin (75 and 67%, respectively) than at a public venue (see Figure 8). In Basel and Bern the opposite was found: in these two cities, 65 and 68% of LPAIs, respectively, took place in state venues (e.g., state-owned community centre, government offices, or government care facilities). While Zurich had the highest disparity of LPAIs taking place at a private venue compared to a state-owned venue (75% vs. 46%, respectively). Overall, the Swiss average of state versus privately owned venues was well-balanced compared to Dublin for which only 33% of LPAIs took place on state-owned premises.

### Duration and frequency

**Figure 9:**
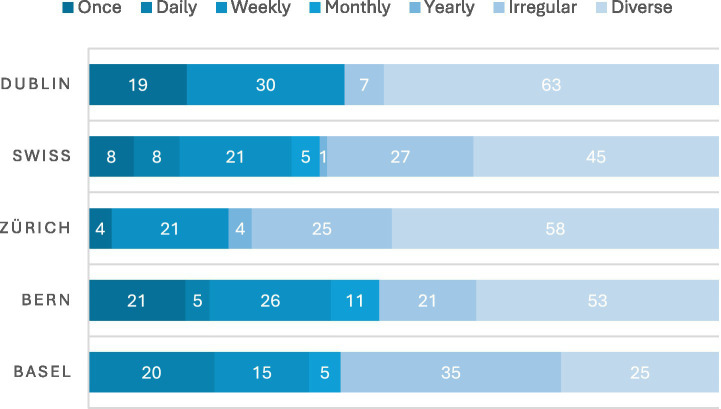
Frequency. Weekly delivery was common (15–30%); Basel had more daily (20%) and irregular (35%) occurring LPAIS, while Zurich/Dublin most often combined regular and irregular contact (58–63%).

A sizable proportion of LPAIs took place weekly (15% of LPAIs in Basel, 26% in Bern, 21% in Zurich, compared to 30% in Dublin) with many other LPAIs taking place irregularly (35% in Basel, 21% in Bern, 25% in Zurich, but just 7% in Dublin; see Figure 9). Often, this was dictated by the participants who might need to access a service on an irregular basis. For example, Bestcompanion (a visiting service) would offer a visiting service for older adults at times that suited them, or Contigo (a service run by the Catholic church), which offered an accompanying service for people who find themselves in difficult situations and would like to feel less lonely. There were a number of one-off interventions, such as the telephonic services Malreden or 143.ch, which participants might access only once. However, the majority of LPAIs took place over diverse frequencies, incorporating both regular and irregular contact. Notably, up to 58% of LPAIs in Zurich and 63% of those in Dublin offered a combination of both regular and irregular formats. Interestingly, Basel bucked this trend, favouring LPAIs that were offered either daily (20%), weekly (15%), or irregularly (35%) (Table 3).

**Table 3:** Duration and frequency.

LPAI/organisation name	Duration of delivery	Frequency of delivery
Basel		
Begegnungszentrum CURA	ST/MT/LT	Daily
Bestcompanion	FL	Irregular
BewegungPlus Basel	FL	Weekly
Caritas	FL	Diverse
Dovida	FL	Diverse
Fundus Basel	ST/MT/LT	Irregular
Grauepanther	FL	Monthly
Häschziit	OO	Irregular
JuBe Basel	ST/OO	Irregular
Jugendzentrum Dreirosen	FL	Daily
M-Eating Table	NS	Irregular
Mein Ohr für Dich	FL/OO	Daily
NotAlone im Quartier	LT	Irregular
Plauderbank	LT	Weekly
Plauderkasse	LT	Daily
Pro Senectute	ST/MT/LT	Diverse
Schweizerisches Rotes Kreuz Basel	FL	Irregular
Treffpunkt Glaibasel	ST/MT/LT	Diverse
ZämmehAlt	ST/MT/LT	Diverse
Zentrum Selbsthilfe	NS	Weekly
Bern		
Caritas	FL	Diverse
Connect! - Together Less Lonely	LT	Irregular
CONTIGO	NS	Irregular
Coontact	FL	Irregular
Der Besuchdienst	FL	Weekly/Diverse
Graue Panther Bern	NS	Daily/Weekly
Heilsarmee	LT	Diverse/Weekly
Helsana	OO	Once
Malreden	OO/LT	Weekly/Once
Nachbarschaft Bern VBG	FL	Diverse
Netzwerk Erzählcafé	FL	Continuous
Offene Kirche Bern	NS	Diverse
Pro Infirmis	FL	Diverse
Pro Senectute Bern	ST/MT/LT	Diverse
Schweizerisches rotes Kreuz Bern	FL/OO	Diverse/Irregular/Once
Selbsthilfe Bern	MT	Monthly/Weekly
Soli Bern	OO/FS	Once/Irregular
Spitex Bern	ST/LT/MT	Diverse
Tavolata	ST/MT/LT	Monthly
Zürich		
147.ch	OO/FL	Irregular
143.ch	OO/FL	Irregular
Anton Schumann Coaching Zurich	FL	Diverse
Arche Zurich	LT	Weekly
Café Yucca	LT	Weekly
Caring Communities	ST/MT/LT	Irregular
Caritas	NS	Diverse
Räber - Coaching & Persönlichkeitsentwicklung	ST/MT	Weekly
Einsamkeit im Alter	OO	Irregular
Gesundheitszentren für das Alter	ST	OO/Continuous
Insieme Zürich	ST/MT/LT/FL/OO	Diverse
Katholisch Kirche Stadt Zürich	ST/OO/FL	Diverse
Netz4	ST/MT/OO/FL	Diverse
Pro Mente Sana	ST/MT/LT/FL/OO	Diverse
Pro Senectute	ST/MT/LT	Once/Weekly/Yearly/Irregular
Psyvita	MT	Diverse
Reformierte Kirche in Zürich	ST/LT/FL/OO	Diverse
Schweizerisches Rotes Kreuz Kanton Zürich	ST/MT/LT/FL/OO	Diverse
Seelsorge.net	ST/MT/LT/FL/OO	Diverse
Selbsthilfe Schweiz	NS	Diverse
Solino	LT/FL	Weekly
Sozialkontakt	FL	Diverse
Spitex Pflege Zürich GmbH	ST/MT/LT/FL/OO	Diverse
Wie geht’s dir?	NS	Irregular
Dublin		
All Ireland Social Prescribing Network	ST/MT/LT	Diverse
Alone.ie	OO/LT	Diverse/Weekly
Archdiocese of Dublin	LT	Weekly
Aware.ie	OO/FL	Diverse
Belongto	ST/MT/LT/OO	Diverse
Childline	OO/FL	Once
City Therapy	MT/LT/FL	Weekly
Community Development Team	LT	Irregular/Diverse
Cross Care	MT/LT/FL	Diverse
Dublin City Community Cooperative	NS	Diverse
Friends of the Elderly	LT/FL/OO	Diverse
Grow Mental Health	MT/FL	Diverse
Haven Hub	ST	Weekly
Jigsaw	FL/OO	Diverse
Making Connections	LT/FL/OO	Diverse
Men’s Sheds	LT/FL	Diverse
Mind and Body Works	MT/FL	weekly
Parentline	OO/FL	Irregular
Samaritans	OO/FL	Once
Seniorline	OO/FL	Once
Shine	OO/FL	Diverse/Once
Social Prescribing	LT/FL	Diverse
SOSAD Ireland	FL/OO	Diverse/Weekly
SpuNout	OO/FL	Once
Barnardos	FL	Diverse
The Crafty Ladies	LT/FL	Weekly
Thrive	FL	Diverse/Weekly

ST, Short-term (0–3 months); MT, Medium-term (up to 1 year); LT, Long-term (more than 1 year); FL, Flexible; OO, Once Off; NS, Not specified.

**Figure 10:**
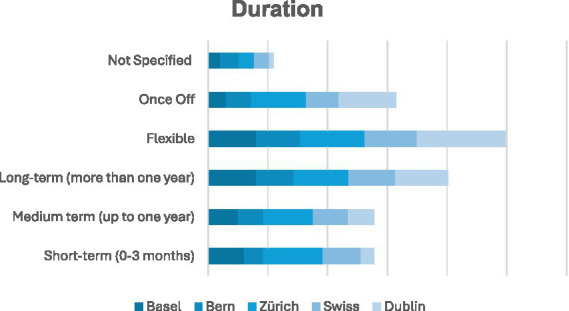
Duration. Many LPAIs are flexible (40% Basel to 74% Dublin) or once-off (15% Basel vs. 46–48% Zurich/Dublin), with 32–46% running up to 1 year.

Considering the large number of irregular or diverse LPAI formats, it is unsurprising that a good proportion of LPAIs (40% in Basel, 32% in Bern, 46% in Zurich, compared to 44% in Dublin) took place over a longer-term (<1 year), were offered on a flexible basis (40% in Basel, 37% in Bern, 54% in Zurich, and 74% in Dublin), or provided as once-off interventions (15% in Basel, 21% in Bern, 46% in Zurich, 48% in Dublin; see Figure 10).

### Types and objectives

Almost three quarters of LPAIs in Swiss cities were focused on some form of support service (75% in Basel, 74% in Bern and 79% in Zurich) while this percentage was higher in Dublin where 93% of LPAIs included or focused on providing support services. Support services were classified as those services that were designed to support participants with particular tasks. For example, Cross Care in Dublin focused on providing housing or welfare support to those in difficult situations, while Pro Senectute offered a range of services including providing temporary administrative support to those who needed it. Many LPAIs (including support services and other types?) were presented in the form of social activities (45% of those in Basel, 32% for Bern, 54% for Zurich, compared with 41% in Dublin). For example, the Jugendzentrum Dreirosen (youth centre Dreirosen) in Basel, offered a place for adolescents to engage with each other in a range of social activities (e.g., playing games), or insieme in Zurich, which hosted a range of social events such as knitting/crocheting groups, or an annual party called ‘inclusions’ where participants could attend and meet others to feel included (Table 4).

**Table 4:** Types and objectives.

LPAI/organisation name	Type of LPAI	Objective of LPAI
Basel		
Begegnungszentrum CURA	SA/SS/TS	SS/SP/SI
Bestcompanion	VA	SI/SP
BewegungPlus Basel	SA/SS/TS	SS/SP/SI
Caritas	SS/TS	SP
Dovida	SS/VA	SP/SI
Fundus Basel	SA/ED/SS/VA	SS/SP/SI
Grauepanther	SA/CE	SI/SP
Häschziit	SH/SS	SP/SI/SC
JuBe Basel	SS/TS	SP
Jugendzentrum Dreirosen	SA/SS	SP/SI
M-Eating Table	SA	SI
Mein Ohr für Dich	SS/TS	SI/SP
NotAlone im Quartier	SS	SP
Plauderbank	SS/CE	SI
Plauderkasse	SS/CE	SI
Pro Senectute	SS/CE/SA/TS	SP/SI
Schweizerisches Rotes Kreuz Basel	VA/SA/TS	SI/SP
Treffpunkt Glaibasel	SS/ED/SA/TS	SP/SI
ZämmehAlt	SS/CA/CE/VA	SP/SI
Zentrum Selbsthilfe	SH	SC/SP/SI
Bern		
Caritas	SS/TS	SP
Connect! - Together Less Lonely	CA	SP/SI/SC
CONTIGO	SS/VA	SP
Coontact	SS/SH	SI
Der Besuchdienst	VA	SP/SI/SC
Graue Panther Bern	SA/CE	SI/SP
Heilsarmee	SS/TS/SA/CE/VA	SP/SI
Helsana	SS	SP
Malreden	TS/SS	SP
Nachbarschaft Bern VBG	CE/SS/SA	SI/SP
Netzwerk Erzählcafé	SS/SH	SS/SI
Offene Kirche Bern	SS/CE/SA	SP/SI
Pro Infirmis	SS/ED/TS	SP/SI
Pro Senectute Bern	SS/CE/SA/VA/TS	SP/SI
Schweizerisches rotes Kreuz Bern	SS/ED/CA/VA	SP/SI
Selbsthilfe Bern	SH	SI/SP/SC
Soli Bern	SS/CA	SS/SI
Spitex Bern	SS/VA	SP/SC
Tavolata	CE/SA	SI
Zürich		
147.ch	SS/TS	SP/SC
143.ch	SS/TS	SP/SC
Anton Schumann Coaching Zurich	TS/SS	SS/SC
Arche Zurich	SA/SS	SP/SI
Café Yucca	SA	SI
Caring Communities	SS/CE/SA	SP/SI
Caritas	SS/TS	SP
Räber - Coaching & Persönlichkeitsentwicklung	TS/SS	SS/SP/SC
Einsamkeit im Alter	SA/ED/CA	SS/SI
Gesundheitszentren für das Alter	SA/SS/TS	SP/SI
Insieme Zürich	SA/SS	SP/SI
Katholisch Kirche Stadt Zürich	SA/SS/TS	SP/SI
Netz4	SA/SS/CE/TS	SI/SP
Pro Mente Sana	TS/SS/CA	SP/SC
Pro Senectute	SA/SS/CE/VA/TS	SP/SI
Psyvita	TS/SS	SC/SS/SP
Reformierte Kirche in Zürich	SA/SS/CE/TS	SP/SI
Schweizerisches Rotes Kreuz Kanton Zürich	SS/CE/VA/TS/SA	SP
Seelsorge.net	TS/SS	SP/SC
Selbsthilfe Schweiz	SH	SC/SP/SI
Solino	SA	SI
Sozialkontakt	SA	SI
Spitex Pflege Zürich GmbH	SS/VA/TS	SP/SI
Wie geht’s dir?	SS/CA/ED	SS/SP
Dublin		
All Ireland Social Prescribing Network	SS/CE/SA	SP/SI
Alone.ie	SS/VA/CA	SP
Archdiocese of Dublin	SS/CE/SA	SP/SI
Aware.ie	SS/TS/ED	SC/SP/SI
Belongto	SA/TS/SS	SP/SI
Childline	SS/TS	SP
City Therapy	SS/TS	SC/SP/SS
Community Development Team	SS/CE	SP/SI
Cross Care	SS/SA/TS	SP/SI
Dublin City Community Cooperative	SS/CE/SA	SP/SI
Friends of the Elderly	SS/SA/VA	SI/SP
Grow Mental Health	SH/SA	SC/SP/SI
Haven Hub	SS/TS	SP/SC
Jigsaw	SS/TS	SC/SP/SS
Making Connections	SA/CE/VA	SI/SP
Men’s Sheds	SS/CE/SA	SI/SP
Mind and Body Works	TS/SS	SI/SC/SS
Parentline	TS/SS	SP
Samaritans	SS/TS	SP
Seniorline	SS/TS	SP
Shine	SS/TS/SH/CE	SP/SI/SC
Social Prescribing	SS/CE/SA	SP/SI
SOSAD Ireland	SS/TS	SS/SP/SC
SpuNout	SS/TS	SP
Barnardos	SS/TS	SP/SI/SC
The Crafty Ladies	SS/CE/SA	SI
Thrive	SS/TS	SC/SP/SS

Type of LPAI: SA, Social Activity; SH, Self-help Group; TS, Therapy (counselling & listening) Services; ED, Educational; SS, Support Service; CA, Campaign; CE, Community Engagement; VA, Visiting and Accompanying Service; Objective of LPAI: SP, Enhance Social Support; SI, Increase Opportunities for Social Interaction; SS, Improve Social Skills; SC, Address Maladaptive Social Cognition.

**Figure 11:**
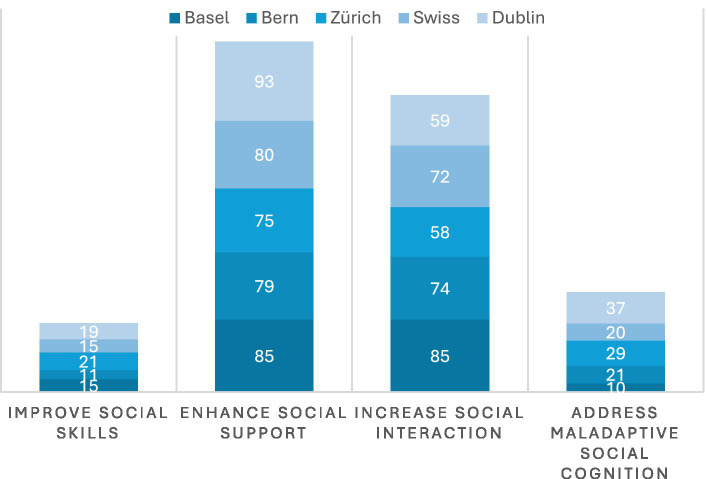
Objective of LPAIs. Objectives concentrated on enhancing social support (75–93%) and social interaction (58–85%), with few LPAIs targeting social skills or maladaptive social cognition.

**Figure 12:**
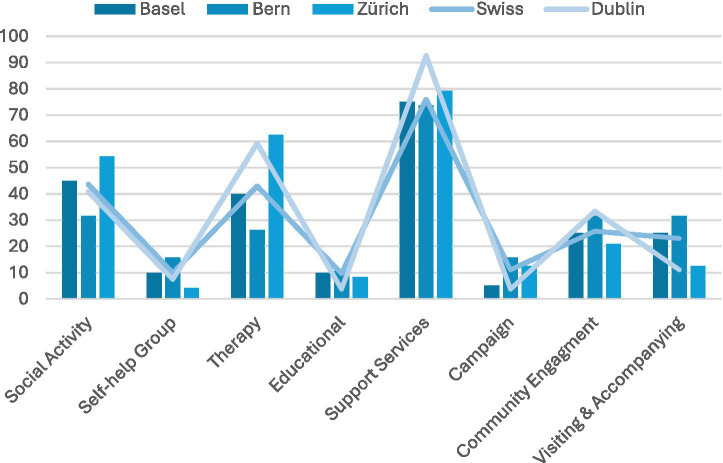
Types of LPAIS. Support services were most prevalent in every city (74–93%), often alongside social activities (32–54%), while self-help and campaigns (e.g., anti-stigma campaigns) were rare (≤16%).

There were a number of therapy groups (often run by private therapy or coaching organisations at a cost), as well as LPAIs that focused on providing accompanying and visiting services (often for a fee). Yet it should be noted that when one considers the number of private therapy practices, the number of those who made any mention of loneliness on their websites was limited (4 in total, 2 in Zurich, 2 in Dublin). Relatively few LPAIs were focused on self-help (10% in Basel, 16% in Bern, 4% in Zurich, compared to 7% in Dublin) or on addressing the stigma of loneliness (5% in Basel, 16% in Bern, 13% in Zurich, compared to 4% in Dublin) (Figures 11, 12).

On the whole, the vast majority of LPAIs aimed to either enhance social support (85% in Basel, 79% in Bern, 75% in Zurich, compared to 93% in Dublin) and/or increase opportunities for social interaction (85% in Basel, 74% in Bern, 58% in Zurich, compared to 59% in Dublin) with only a few focused on improving social skills, or addressing maladaptive social cognition through therapy, education, or coaching. Importantly, very few LPAIs followed a formal methodology for either alleviating or preventing loneliness (11% average in Switzerland—mainly concentrated in private therapy practices) although a large number did include loneliness as part of their organisational reporting/self-evaluation (80% in Basel, 68% in Bern, 83% in Zurich, compared to 63% in Dublin).

**Figure 13:**
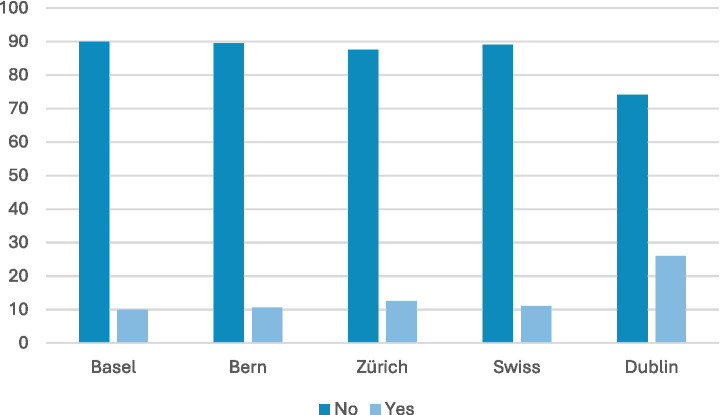
Follows a formal methodology or evaluation. Only 10–13% of Swiss LPAIs followed a formal loneliness methodology (vs 26% in Dublin).

**Figure 14:**
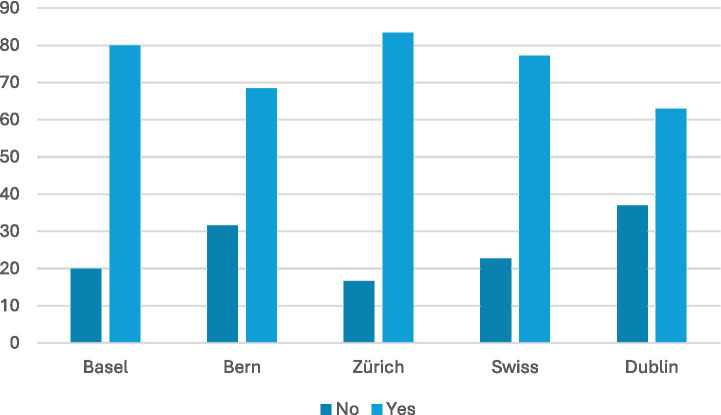
Reported evaluation/self-monitoring. Most LPAIs reported some form of effectiveness monitoring/evaluation (approximately 63–83% across cities).

Very few LPAIs in Switzerland followed a formal methodology (10% in Basel, 11% in Bern, 13% in Zurich; see Figure 13) while the percentage was higher in Dublin (26%). However, a high proportion of interventions did report an evaluation of their services (80% in Basel, 68% in Bern, 83% in Zurich compared to 68% of Dublin; see Figure 14).

### Social relationship expectation score

**Figure 15:**
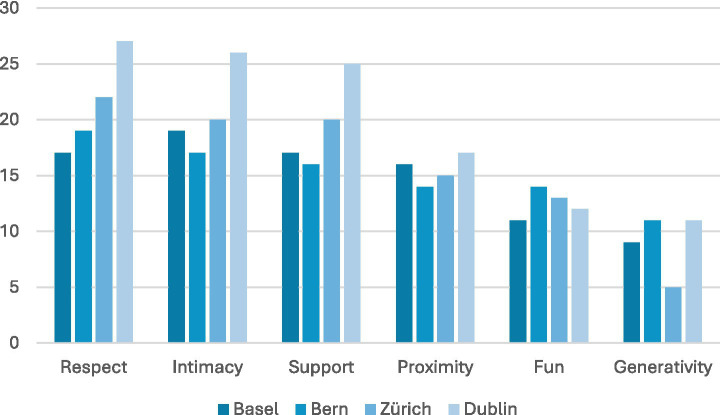
SRE score by city. All aspects of the SRE score were well represented. Dublin scored highest overall out of 27 LPAIs 27 included aspects of Respect, 26 Intimacy, and 25 Support.

All six aspects of the Social Relationship Expectation (SRE) framework (44) were well represented among the LPAIs surveyed, with Respect, Intimacy, and Support being particularly prominent (see Figure 15). In contrast, the aspect of Fun (11 LPAIs in Basel, 14 in Bern, and 13 in Zurich, compared to 12 in Dublin) and Generativity (having opportunities to contribute meaningfully to others) were less well represented (9 in Basel, 11 in Bern, 5 in Zurich, compared to 11 in Dublin). Here, only 36 and 50 out of the 90 included LPAIs offered services that addressed participant’s desire for fun (e.g., The Crafty Ladies in Dublin) or generativity (e.g., Caring Communities in Zurich) respectively. This distribution was further evident when broken down by city, with all surveyed cities displaying similar weightings towards Respect, Support, Intimacy, and Proximity, and lower representation of Fun and Generativity.

**Figure 16:**
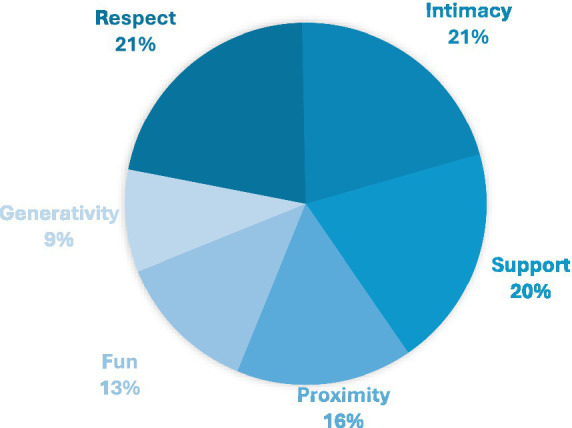
SRE prevalence across all cities. All aspects of the SRE score were well represented with respect (21%), intimacy (21%) and support (20%) dominating overall; proximity was moderate (16%), while Fun (13%) and Generativity (9%) were least prevalent across all cities.

Dublin stood out as a strong performer, offering the highest number of services addressing the most SRE aspects (see Figure 16). In particular, almost all LPAIs in Dublin addressed the aspects of Respect (27/27), Intimacy (26/27), and Support (25/27). Even while Generativity was generally weak across the cities surveyed, in Dublin it was found to be present in 11 out of 27 LPAIs. Following Dublin, Zurich was evaluated to have a high satisfaction in core aspects, although it too had a large gap for Fun and Generativity. Basel was the weakest of the four cities in relational fulfilment scoring, although it scored well in Intimacy and Support, with 19 and 17 LPAIs, respectively, supporting these aspects out of a total of 20 LPAIs included for Basel in our survey (Table 5).

**Table 5:** Social relationship expectation score.

LPAI/organisation name	SRE					
	Proximity	Support	Intimacy	Fun	Generativity	Respect
Basel						
Begegnungszentrum CURA	Yes	Yes	Yes	Yes	Yes	Yes
Bestcompanion	Yes	Yes	Yes	Yes	No	No
BewegungPlus Basel	Yes	Yes	Yes	Yes	Yes	Yes
Caritas	No	Yes	Yes	No	No	Yes
Dovida	Yes	Yes	Yes	Yes	No	Yes
Fundus Basel	Yes	Yes	Yes	Yes	No	Yes
Grauepanther	Yes	No	Yes	Yes	Yes	Yes
Häschziit	Yes	Yes	Yes	No	Yes	Yes
JuBe Basel	No	Yes	Yes	No	No	Yes
Jugendzentrum Dreirosen	Yes	Yes	Yes	Yes	Yes	Yes
M-Eating Table	Yes	No	No	Yes	No	No
Mein Ohr für Dich	No	Yes	Yes	No	No	Yes
NotAlone im Quartier	Yes	Yes	Yes	No	Yes	Yes
Plauderbank	Yes	No	Yes	No	Yes	Yes
Plauderkasse	Yes	Yes	Yes	No	No	Yes
Pro Senectute	Yes	Yes	Yes	Yes	Yes	Yes
Schweizerisches Rotes Kreuz Basel	Yes	Yes	Yes	Yes	No	Yes
Treffpunkt Glaibasel	No	Yes	Yes	No	No	No
ZämmehAlt	Yes	Yes	Yes	Yes	No	Yes
Zentrum Selbsthilfe	Yes	Yes	Yes	No	Yes	Yes
Bern						
Caritas	Yes	Yes	Yes	Yes	No	Yes
Connect! - Together Less Lonely	Yes	Yes	Yes	No	No	Yes
CONTIGO	No	Yes	Yes	No	No	Yes
Coontact	Yes	No	Yes	Yes	Yes	Yes
Der Besuchdienst	Yes	Yes	Yes	Yes	No	Yes
Graue Panther Bern	Yes	Yes	Yes	Yes	Yes	Yes
Heilsarmee	Yes	Yes	Yes	Yes	Yes	Yes
Helsana	No	Yes	No	No	No	Yes
Malreden	No	Yes	Yes	Yes	No	Yes
Nachbarschaft Bern VBG	Yes	Yes	Yes	Yes	Yes	Yes
Netzwerk Erzählcafé	Yes	No	Yes	Yes	Yes	Yes
Offene Kirche Bern	Yes	Yes	Yes	Yes	Yes	Yes
Pro Infirmis	No	Yes	Yes	No	No	Yes
Pro Senectute Bern	Yes	Yes	Yes	Yes	Yes	Yes
Schweizerisches rotes Kreuz Bern	Yes	Yes	Yes	Yes	Yes	Yes
Selbsthilfe Bern	Yes	Yes	Yes	Yes	Yes	Yes
Soli Bern	Yes	No	No	Yes	Yes	Yes
Spitex Bern	No	Yes	Yes	No	No	Yes
Tavolata	Yes	Yes	Yes	Yes	Yes	Yes
Zürich						
147.ch	No	Yes	Yes	No	No	Yes
143.ch	No	Yes	Yes	No	No	Yes
Anton Schumann Coaching Zurich	No	Yes	Yes	No	No	Yes
Arche Zurich	Yes	Yes	Yes	Yes	No	Yes
Café Yucca	Yes	Yes	Yes	Yes	No	Yes
Caring Communities	Yes	Yes	Yes	No	Yes	Yes
Caritas	No	Yes	Yes	No	No	Yes
Räber - Coaching & Persönlichkeitsentwicklung	No	Yes	Yes	No	No	Yes
Einsamkeit im Alter	Yes	No	Yes	Yes	No	Yes
Gesundheitszentren für das Alter	Yes	Yes	No	Yes	No	Yes
Insieme Zürich	Yes	Yes	Yes	Yes	No	Yes
Katholisch Kirche Stadt Zürich	Yes	Yes	Yes	Yes	Yes	Yes
Netz4	Yes	Yes	Yes	Yes	No	Yes
Pro Mente Sana	No	Yes	Yes	No	No	Yes
Pro Senectute	Yes	Yes	Yes	Yes	No	Yes
Psyvita	Yes	Yes	Yes	No	Yes	Yes
Reformierte Kirche in Zürich	Yes	Yes	Yes	Yes	Yes	Yes
Schweizerisches Rotes Kreuz Kanton Zürich	No	Yes	Yes	No	No	Yes
Seelsorge.net	No	Yes	Yes	No	No	Yes
Selbsthilfe Schweiz	Yes	Yes	Yes	Yes	Yes	Yes
Solino	Yes	No	Yes	Yes	No	Yes
Sozialkontakt	Yes	No	No	Yes	No	No
Spitex Pflege Zürich GmbH	Yes	Yes	Yes	Yes	No	Yes
Wie geht’s dir?	No	Yes	No	No	No	Yes
Dublin						
All Ireland Social Prescribing Network	Yes	Yes	Yes	Yes	Yes	Yes
Alone.ie	Yes	Yes	Yes	No	No	Yes
Archdiocese of Dublin	Yes	Yes	Yes	Yes	No	Yes
Aware.ie	Yes	Yes	Yes	No	No	Yes
Belongto	Yes	Yes	Yes	Yes	No	Yes
Childline	No	No	Yes	No	No	Yes
City Therapy	No	Yes	Yes	No	No	Yes
Community Development Team	Yes	Yes	No	Yes	Yes	Yes
Cross Care	Yes	Yes	Yes	Yes	No	Yes
Dublin City Community Cooperative	Yes	Yes	Yes	Yes	Yes	Yes
Friends of the Elderly	Yes	Yes	Yes	Yes	Yes	Yes
Grow Mental Health	Yes	Yes	Yes	Yes	Yes	Yes
Haven Hub	No	Yes	Yes	No	No	Yes
Jigsaw	Yes	Yes	Yes	No	Yes	Yes
Making Connections	Yes	Yes	Yes	Yes	Yes	Yes
Men’s Sheds	Yes	Yes	Yes	Yes	Yes	Yes
Mind and Body Works	No	Yes	Yes	No	No	Yes
Parentline	No	Yes	Yes	No	No	Yes
Samaritans	No	Yes	Yes	No	No	Yes
Seniorline	No	Yes	Yes	No	No	Yes
Shine	Yes	Yes	Yes	No	No	Yes
Social Prescribing	Yes	Yes	Yes	Yes	Yes	Yes
SOSAD Ireland	No	Yes	Yes	No	No	Yes
SpuNout	No	No	Yes	No	No	Yes
Barnardos	Yes	Yes	Yes	No	Yes	Yes
The Crafty Ladies	Yes	Yes	Yes	Yes	Yes	Yes
Thrive	No	Yes	Yes	No	No	Yes

## Discussion

The data presented here reveals both encouraging and challenging findings regarding the state of LPAIs available to the public. On the one hand, it is encouraging to see that compared to other surveys, our survey indicates that the Swiss population is better served than elsewhere. For example, the JRC EU survey revealed 2.7 interventions per million inhabitants for Northern-EU states (43), while Duncan et al.’s Survey indicated 2.66 interventions per million inhabitants in England. Swiss cities surveyed here had on average 86.3 interventions per million. Nevertheless, Swiss cities, do not appear to be as well served as other cities. Barcelona’s municipal survey, for example, indicated 157.65 interventions per million in habitants (38). It is noteworthy that the present study indicates 19.29 interventions per million inhabitants in Dublin, far off the JRC EU estimate of 2.54 (43). Some of the discrepancies may be accounted for by differences in methodologies. For example, the present study included interventions that expressly sought to alleviate and prevent loneliness while the JRC EU study focused solely on alleviating loneliness. Nevertheless, even using the same mapping approach, intervention density was substantially higher in Swiss cities than in Dublin suggesting that—at least in terms of explicitly loneliness-framed provision—Dublin residents may face comparatively smaller options for alleviating or preventing loneliness.

While the number of LPAIs available to the public is encouraging, there are important gaps in the way LPAIs are operationalised. We may highlight five important gaps.

### Structural gaps and institutional responsibility

Across all cities surveyed, NGOs emerged as the dominant providers of LPAIs, accounting for 85% of offerings in Switzerland and as much as 89% in Dublin. In contrast, direct state administration of LPAIs was negligible, this is despite public venues regularly being used for delivery (e.g., 65% in Basel, and 68% in Bern). This is in stark contrast to the JRC EU mapping which indicated that as few as 37% of NGOs were responsible for loneliness alienation interventions (43). Even taking into account the slight difference in methodology between the studies, the data presented here indicates an excessively high reliance on NGO’s for LPAIs. In addition, while the state is supplying some funding to more than 60% of LPAIs in Switzerland, and about 75% in Dublin, the precise scale and consistency of state funding is difficult to ascertain without in-depth analysis of the financial records of each intervention, an activity that was beyond the scope of this study. Nevertheless, our data suggests that, in contrast to the Swiss context, LPAIs in Dublin are more likely to receive state funding (75%) while at the same time there are les state-organised LPAIs. This suggests a slightly stronger ‘state as funder but not provider’ model than in Swiss cities. In both cases, however, the context may create particular vulnerabilities in LPAI offerings as capacity remains concentrated in NGOs rather than being embedded in public services. This context is exasperated by the discrepancy in place-based delivery between Dublin and Swiss cities. Dublin relied less on state premises (33%) than Basel (65%) or Bern (68%) suggesting few public-sector connections where loneliness support can be administered.

This uncertainty in funding models represents a risk to equitable access to LPAIs and their sustainability. This is particularly true in Dublin where funding is marginally less diversified than in Swiss cities (e.g., higher state funding, 56% vs. 65% for corporate/foundation support, and 74% vs. 80% for public donations). Our survey demonstrates that many interventions are offered free of charge. However, those that did impose costs were often prohibitively expensive, especially in Dublin whose cost profile appears more polarised than in Swiss cities. These high costs can disproportionately exclude those who would benefit most from such services. For example, at risk groups such as migrants, adolescents, or those who are chronically ill. These population segments are often economically at a disadvantage. Considering that psychological interventions (such as cognitive behaviour therapy (CBT), or coaching to address maladaptive social cognition) has been shown to be most effective in alleviating loneliness (12, 29), their equitable accessibility should be a policy priority, not left to the free market.

Taken together, the combination of (i) a small pool of methodologically ‘formal’ interventions and (ii) their tendency to sit in the higher cost bands, indicates a potential equity gap in access to higher-intensity support. With limited state administered LPAIs, and no clear funding strategy, the state (at least in Switzerland and Ireland) is acting in a supportive role rather than leading the fight against loneliness. This limited visibility on the part of the state risks undercutting long-term strategic planning. This is surprising considering our introductory remarks, which have argued that loneliness is a serious health concern with causal links to decreased mental and physical health outcomes. Loneliness’ causal link to morbidity and mortality comparable to smoking or obesity (11, 14), should merit a response akin to the government’s responses to smoking, drinking, drug abuse, or obesity. Given that loneliness—especially in higher income context such as Switzerland and Ireland—is on the increase, it is imperative that governments consider the effectiveness, or indeed the ethics, of outsourcing LPAIs to under-resourced civil society actors.

### Gaps for at-risk populations

It is well-established that loneliness research disproportionately focuses on older adults, something that is slowly beginning to change as more and more research is conducted. The results of our survey are comparable to other similar surveys and indicates that this focus is present not only in theoretical research but as well in how LPAIs practically attempt to address loneliness in society. Our survey demonstrated that as much as 45% of interventions are orientated directly towards older adults and one may reasonably presume that those interventions focused on the general adult population also include a focus on older adults. This is a similar to the results of the JRC EU mapping (55%) (43), as well as a Scottish survey of befriending services (37). Other at-risk groups, such as adolescents and middle-aged adults, remain underserved. The JRC EU map indicates as little as 8% of interventions target youth and expressly call for an increase in this. This reflects a policy blind spot, especially when one considers that loneliness in youth has risen markedly in the last two decades (50, 51). While we recognise that a small number of dynamic youth interventions exist (e.g., 147.ch, Jugendzentrum Dreirosen, and Häschziit), the overall landscape in Switzerland does not reflect the full spectrum of those at risk.

The underrepresentation of at-risk populations groups includes those with disabilities and chronic illness—despite robust evidence pointing to these groups being disproportionately affected by loneliness (52)—as well as marginalised communities such as immigrants or LGBTQAI+ individuals. This is similar to the findings of other reports where LGBTQAI+ groups and those living with disabilities were particularly poorly served (37, 53). Although it should be noted that in our survey there were examples of LPAIs that had such population groups in mind, for example, “Belong To” the national LGBTQ+ youth organisation in Ireland. Yet LPAIs with a clear focus on these groups were not widely present, and certainly not in every city surveyed. A more precise needs assessment and equity-orientated planning of LPAI development and deployment is lacking.

### Gaps in design and delivery modalities

As briefly noted above, although there is good evidence that support focusing on the individual, such as CBT, or coaching, can alleviate loneliness, these LPAIs are expensive and can exclude at-risk populations that may not be able to afford them. There is substantial evidence suggesting that group interventions are more effective than individual-based interventions where these groups are small enough to allow for trust and discussion, but large enough for dynamic interaction and the development of multiple relational possibilities (around 10 participants) (30, 54). For these groups to be effective, the relationships formed need to be meaningful. An analysis of our data, however, indicates that this may not be the case. Our data suggests a broad preference for interventions focused on individual-level support or for groups that are larger (above 10). Many LPAIs were focused on the individual (e.g., providing support services to individuals), with other LPAIs—particularly those focused on social activities—forming larger groups (above 10) around activities that do not necessary result in meaningful relationships. For example, Graue Panther Bern that held once off events (e.g., talks, concerns, outings to interesting places etc.) which its members could attend. These groups fluctuate greatly in terms of size and composition and would not be conducive to forming meaningful relationships.

The use of between-session interaction (through practice, facilitator contact and/or group contact with other participants) increases the likelihood of an intervention to be successful on the long term (54). Interventions that include a learning mechanism to improve friendship or community connection (i.e., opportunities to learn about behavioural change techniques, friendship or community connection, and/or health education) tend to yield positive intervention outcomes too (54, 55). While there is no consensus regarding the efficacy of interventions aimed at enhancing social support (29, 56), interaction through active participation (in terms of mindfulness exercises, physical exercise, community or social events, and/or specific assignments) and group or facilitator contact (including memory/shared experience, group discussion, group session practice, facilitator feedback, and online or phone messaging) appear to be beneficial (54). Psychological interventions and/or interventions that include cognitive components have been shown to provide the most convincing evidence for reducing loneliness (12, 31, 55), especially those addressing maladaptive social cognitions (29). Our survey, however, identified no LPAI that sought to form groups of about 10 participants so as to foster meaningful relationships and incorporate these established practices with a focus on preventing or alleviating loneliness. This is a serious gap that should be addressed.

It is interesting to note that very few of the interventions identified in this survey followed a formal methodology. Of those that did, these were primarily professional services (e.g., counselling services). By contrast, a large portion of LPAIs did publish annual reports on their websites that included basic evaluation metrics for their interventions (e.g., how many participants engaged in their services) but not on measuring the impact of their interventions on loneliness. This appears contradictory; in the absence of a formal methodology concrete success metrics are difficult to determine. This challenge was faced by a UK government survey on loneliness (53). In this survey two important barriers to measuring loneliness were identified: 1) LPAI providers were concerned that asking questions about loneliness for measurement purposes could alienate individuals because of the stigma associated with loneliness. 2) LPAI providers felt that measures were not seen as being sensitive to the lived-experience of loneliness where it was unrealistic to view single interventions as having an impact on loneliness, or where factors other than interventions can negatively influence the subjective experience of loneliness. Thus LPAI providers perceived loneliness measurements as being of limited use and ‘at odds with core intervention values of being person-centred and response to participant needs.’ (53) This raises serious questions about the suitability of reports produced by LPAI providers. One can assume that such reporting is likely intended to satisfy funding conditions or justifying further funding. This, however, risks functioning more as a symbolic exercise than a genuine effort to assess the effectiveness of their interventions. In this we join with others, such as the Welsh government (57), calling for more (responsibly implemented) technological support to alleviate loneliness generally and in particular in Switzerland.

### Gaps in the use of technology or innovation

A noticeable finding based on our data was the underutilisation of modern technology, particularly in Swiss cities. It is true that telephone-based LPAIs were present (e.g., 143.ch, Malreden, or Mein Ohr Für Dich) and that Dublin demonstrated a high percentage of virtually accessible LPAIs (78%). This is very much in line with other surveys (37, 53). Yet very few examples of Swiss or Irish LPAIs were leveraging innovative digital tools, such as mobile applications or integrated web platforms. Exceptions were Häschziit—which encouraged participants to share their questions, concerns, or thoughts on an innovative platform for teenagers and young adults aged 17 + − and Wie geht’s dir? (How are you)—a web-based platform for boosting mental health. This is particularly striking when one considers the proliferation of eHealth tools and their proven potential to overcome geographic and psychological barriers to engagement.

Indeed, there are mobile applications dedicated to preventing or alleviating loneliness—e.g., the Loneliness App (available on Google Play) or the Loneliness Test and CT: Loneliness (available on Apple’s App Store). Yet this technology remains disconnected from the local communities represented in the cities survey. The evidence is mixed as to the effectiveness of technological tools for combating loneliness, yet evidence suggests its effectiveness may depend more on how the technology is used (58). Importantly, technology should never be used as a replacement for in-person connection (53, 57). Rather it may be used as a scaffold to support in-person connect. In this regard, other countries, such as the UK are showing more purpose-built digital scaffolding (e.g., apps to arrange meetings or social skills practice apps) (53) for personal connections than Switzerland or Ireland—even if exceptions are notable. This represents an innovation gap whereby LPAIs are failing to leverage the rise of new technologies to prevent or alleviate loneliness. It is possible this is a direct consequence of the informal, ad-hoc nature of LPAIs in the cities surveyed. Without a clear state strategy or resources, small, isolated NGOs struggle to use new technologies effectively.

### Gaps in relational depth

The application of the SRE framework (44) to our data reveals a consistent emphasis on foundational aspects of social connection (Respect, Support and Intimacy) and an underemphasis on more agentic and dynamic components like Fun and Generativity. While 85 out of 90 LPAIs addressed Respect and 82 addressed Intimacy, only 36 addressed the aspect of Generativity. That is to say, only 36 LPAIs actively encouraged participants to actively participate, or take responsibility for an LPAI. This is interesting considering the large number of interventions identified within our research and other studies that focus on social activities. In our study up to 85% of interventions in Basel focus on social interaction, with a Swiss average of 74%, while in the JRC EU report 41% of interventions focus on connecting people with an additional 26% on social activities (43). While LPAIs, on the whole, focus on bringing people together, they do so with a view to participants as passive consumers, rather than active contributors. This suggests that current LPAIs may be reinforcing dependence rather than promoting mutual engagement or active contribution. Generativity is not a superficial luxury; it is a core dimension of a fulfilling social life and a key buffer against subjective feelings of loneliness. LPAIs that enable participants to contribute and derive joy through active engagement are more likely to have enduring effects. There is, therefore, a need for LPAIs to explicitly evaluate their ability to focus on the lesser-addressed relational expectation of Generativity or Fun.

Applying the SRE framework to LPAIs is one way to evaluate an important aspect of the suitability of an LPAI to meet the subjective desired relational expectations of participants. Dublin outperformed Swiss cities in broadly addressing SRE aspects. For example, all 27 Dublin-based LPAIs addressed Respect, with 26 addressing Intimacy. Even Generativity (an underrepresented aspect in our survey) was addressed in 11 Dublin LPAIs compared to a Zurich (with only five). It is possible that these differences reflect variations in the prioritisation of community-driven metrics within Irish culture, but it may also arise from the lack of a clear loneliness strategy or suitable resources to operationalise empirically established strategies to maximise the effectiveness of LPAIs.

## Limitations

Several limitations should be noted. First, there remains a significant stigma associated with loneliness. It is highly likely that some LPAIs exist which do not explicitly mention loneliness on their websites or social media platforms, for fear of dissuading potential participants. Consequently, a number of active LPAIs may have been excluded from our research. This limitation has been identified in other studies, such as the JRC EU’s mapping of loneliness interventions (43). To address this limitation, we engaged in both purposive and snowball sampling by approaching key members of the community (see methodology above) who may have knowledge of relevant LPAIs, and by asking survey respondents to recommend other LPAIs. However, less than one third of those contacted completed the survey and many key community members did not respond to our inquiries. This even with reminder emails being sent to all LPAIs and key members. Among LPAIs mentioned by key members, many had been already identified in our semi-structured search.

Second, developing a clear, consistent cataloguing methodology for LPAIs proved to be difficult—a limitation shared with other studies, such as the JCR EU’s mapping (43). This is partly because of the stigma associated with loneliness mentioned above, and partly as a result of the lack of clear strategy for addressing loneliness evidenced by the convoluted structure within which LPAIs are operationalised. While some organisations/LPAIs were overtly focused on loneliness (e.g., Einsamkeit im Alter—Loneliness in Old Age), others mentioned loneliness only as part of a broader strategy with many preferring to reference loneliness only in their annual reports aimed primarily at funders or internal memberships. A major challenge was the complex structures at play. Some LPAIs acted independently (e.g., private organisations, such as Soli or Anton Schumann Coaching), other LPAIs were semi-autonomous, having a dedicated website but being part of larger organisations/networks, such as Café Yucca, which was heavily supported by both the reformed and catholic churches of Switzerland (both of which are independently included in our survey). Other organisations had multiple activities that may, or may not, address loneliness and yet their over-arching organisation reported on loneliness across all their activities (e.g., Pro Senecute). Some LPAIs were included in networks dedicated to community well-being (e.g., Fundus), sometimes these networks themselves came under the umbrella of other institutions (e.g., Connect is heavily supported by the Public Health Services of Switzerland). In other cases, larger national organisations had local offices in different cities (e.g., Caritas, The Swiss Red Cross, or the larger Christian denominations) where LPAIs were operationalised either semi-autonomously, as part of the city office, or under the umbrella of the national organisation.

Our methodology sought to treat LPAIs as independently as possible, but also to recognise larger organisations with many activities, some of which may be LPAIs. It is, therefore, possible that some LPAIs were included twice (under their own name, but also included in reporting on their umbrella organisation). The complexity of the situation makes it impossible to identify every single LPAI independently of its wider networks/umbrella organisations. While this should be borne in mind when interpreting our findings, it is important to note that our reporting here is descriptive. We have sought to be as consistent as possible across the cities surveyed so as to produce an ‘as accurate as possible’ comparative analysis of a highly complex context.

Third, our data is limited to urban centres in two high-income countries and in particular to the largest German speaking cities of Switzerland. This is important because Switzerland has a cantonal system with significant differences between the 26 cantons. Our survey focused on three of the largest cantons, and it is likely that differences may be present in other language regions or among the smaller cantons. Limited resources meant that the research was not able to survey other large language regions of Switzerland. Rural areas or lower-income contexts, where loneliness may be compounded by geographic isolation or a lack of services, were not included in this research. Future research surveying LPAIs would do well to examine these important aspects.

## Conclusions

In light of our findings, we recommend three major shifts in policy. First, governments must take an increasingly proactive role. Beyond merely supporting independent NGOs with limited ad-hoc funding or access to government premises, loneliness should be treated as a serious public health issue, on par with smoking, drinking, or obesity. This entails producing a clear strategy, supplying adequate resources (including funding) as well as initiating, coordinating, and earnestly evaluating the effectiveness of LPAIs. The current NGO-centric, public-donation funded model is insufficient to meet the rising prevalence of loneliness. State leadership is needed to set standards, ensure resources, and integrate loneliness prevention and alleviation into broader health and social care strategies.

Second, equitable access to LPAIs must be safeguarded. Given the empirical research into the efficacy of certain types of LPAIs (particularly psychological and cognitive interventions) which tend to be higher-cost services—and that our results demonstrated these services were mostly offered by private organisations - those that address these aspects of loneliness should be universally accessible. Policymakers should consider mechanisms (e.g., public health reimbursement schemes) to protect equitable access to evidence-informed, higher-intensity support. Loneliness should be seen not merely as a social issue but as a public health concern warranting resources akin to those deployed to address smoking, drinking, or obesity.

Third, Innovation (and with it, earnest evaluation) must be incentivised. State and private funders should prioritise supporting innovative LPAIs that incorporate both well-established models (e.g., face-to-face small groups that promote meaningful relationships) as well as new technologies such as mobile AI based platforms that connect in-person groups and virtual communities in ways that incorporate the full spectrum of participants social expectations.

This study reveals that while Swiss and Irish cities offer a rich tapestry of LPAIs, these interventions often fall short of addressing the multidimensional nature of loneliness, especially when considered in light of the SRE framework (44). Existing interventions disproportionately favour passive support over participatory engagement, overlook high-risk but underserved groups, and fail to exploit digital technologies. If we are to tackle the pandemic that has become loneliness, it is imperative that state actors make urgent policy shifts that provide clear direction and leadership.

## Data Availability

The original contributions presented in the study are included in the article/supplementary material, further inquiries can be directed to the corresponding author.

## Footnotes

1 This applied even if an intervention was originally initiated to combat loneliness (or had even completed a survey claiming loneliness as a focus) but no discernible focus on loneliness could be found on their website/literature. For example, the founder of the Facebook group ‘Zurich Together’ told a local newspaper that they initiated the social media group because they felt lonely and wanted to connect with others who felt the same. The group has more than two thousand followers, but nowhere on the group page is loneliness mentioned, nor is there any clear indication that loneliness is a concern for the group (59).

## Appendix: search string

German

Basel [Bern/Zürich/Dublin] OR Einsam* OR Allein* OR Einzeln OR Vereinzelt OR Kontaktlos OR Gemeinschaft OR Gesellschaft* OR Verbundenheit OR Verbindung OR Inklu* OR Teilhabe OR Sozial OR Soziale Aktivität OR Soziale Unterstützung OR Intervention OR Gruppe OR Netzwerk OR Verein* OR Aktivität OR Organisation OR Programm OR Bund OR Klub OR Sozialer Kreis OR Stiftung OR Verband OR Assoziation OR Projekt OR Hilfe OR Unterstützung.

English

Basel [Bern/Zurich/Dublin] OR lonely* OR alone* OR single OR isolated OR contactless OR community OR society* OR connectedness OR connection OR inclusion* OR participation OR social OR social activity OR social support OR intervention OR group OR network OR association* OR activity OR organization OR program OR federation OR club OR social circle OR foundation OR union OR association OR project OR help OR support.
